# Comparative analysis of stress distribution in one-piece and two-piece implants with narrow and extra-narrow diameters: A finite element study

**DOI:** 10.1371/journal.pone.0245800

**Published:** 2021-02-04

**Authors:** Fabricia Teixeira Barbosa, Luiz Carlos Silveira Zanatta, Edélcio de Souza Rendohl, Sergio Alexandre Gehrke

**Affiliations:** 1 Department of Implantology, Paulista University, São Paulo, Brazil; 2 Department of Dentistry, Santo Amaro University, São Paulo, Brazil; 3 Department of Biotechnology, Universidad Católica de Murcia, Murcia, Spain; 4 Department of Research, Biotecnos—Technology and Science, Montevideo, Uruguay; Virginia Commonwealth University, UNITED STATES

## Abstract

**Objectives:**

The aim of this in vitro study was to evaluate the stress distribution on three implant models with narrow and extra-narrow diameters using the finite element method (FEA).

**Materials and methods:**

Dental implants of extra-narrow diameter of 2.5 mm for a one-piece implant (group G1), a narrow diameter of 3.0 mm for a one-piece implant (group G2) and a narrow diameter of 3.5 mm for a two-piece implant with a Morse taper connection (group G3). A three-dimensional model was designed with cortical and cancellous bone, a crown and an implant/abutment set of each group. Axial and angled (30°) loads of 150 N was applied. The equivalent von Mises stress was used for the implants and peri-implant bone plus the Mohr-Coulomb analysis to confirm the data of the peri-implant bone.

**Results:**

In the axial load, the maximum stress value of the cortical bone for the group G1 was 22.35% higher than that the group G2 and 321.23% than the group G3. Whereas in angled load, the groups G1 and G2 showing a similar value (# 3.5%) and a highest difference for the group G3 (391.8%). In the implant structure, the group G1 showed a value of 2188MPa, 93.6% higher than the limit.

**Conclusions:**

The results of this study show that the extra-narrow one-piece implant should be used with great caution, especially in areas of non-axial loads, whereas the one- and two-piece narrow-diameter implants show adequate behavior in both directions of the applied load.

## Introduction

Frequently, areas with anatomical limitations of bone thickness or mesio-distal clinical space (interradicular space) are found to replacement of teeth less. In these cases, the use of small diameter implants may be an alternative [1,2] to the surgery for increase the bone volume or to orthodontic movement for open the mesio-distal space. Mechanically, small diameter implants are less resistant than larger diameter implants [1,3] and have a smaller contact surface, a factor that directly influences the transfer of forces to the peri-implant bone, which can compromise success long-term. However, the use of small diameter implants has shown survival rates similar to standard diameter implants [4–7].

Small-diameter implants have been modified so that their fracture resistance and force dissipation are improved. However, when these implants are manufactured in two parts, the presence of an internal coupling chamber for the abutment and the fixation screw makes it difficult to reduce the diameter of this implant without affecting its strength. As a result, some companies started to manufacture small-diameter one-piece implants (implant and abutment), creating a more resistant structure [8].

Some methodologies, such as finite element analysis and photoelasticity, have been used in order to evaluate the behavior of implants since an adequate distribution of the forces applied during the masticatory function is fundamental for the long-term success of implant-supported rehabilitation [9,10]. In this sense, a finite element analysis allows for an analytical assessment of the distribution of forces through a virtual mathematical model, where variables can be tested.

The use of one-piece narrow-diameter implants to avoid previous procedures (grafts and/or orthodontic movement) brings an immediate benefit to the solution of the case. However, other complications in the medium and long term, mainly due to the biomechanical behavior of these implants, can affect the durability of the treatment. In this sense, the present in vitro study aimed to apply the method of finite elements to assess and compare the behavior of the concentration and distribution of a load of 150 N, applied in the axial and oblique directions, on extra-narrow- and narrow-diameter one- and two-piece implants.

## Materials and methods

**Fig 1:**
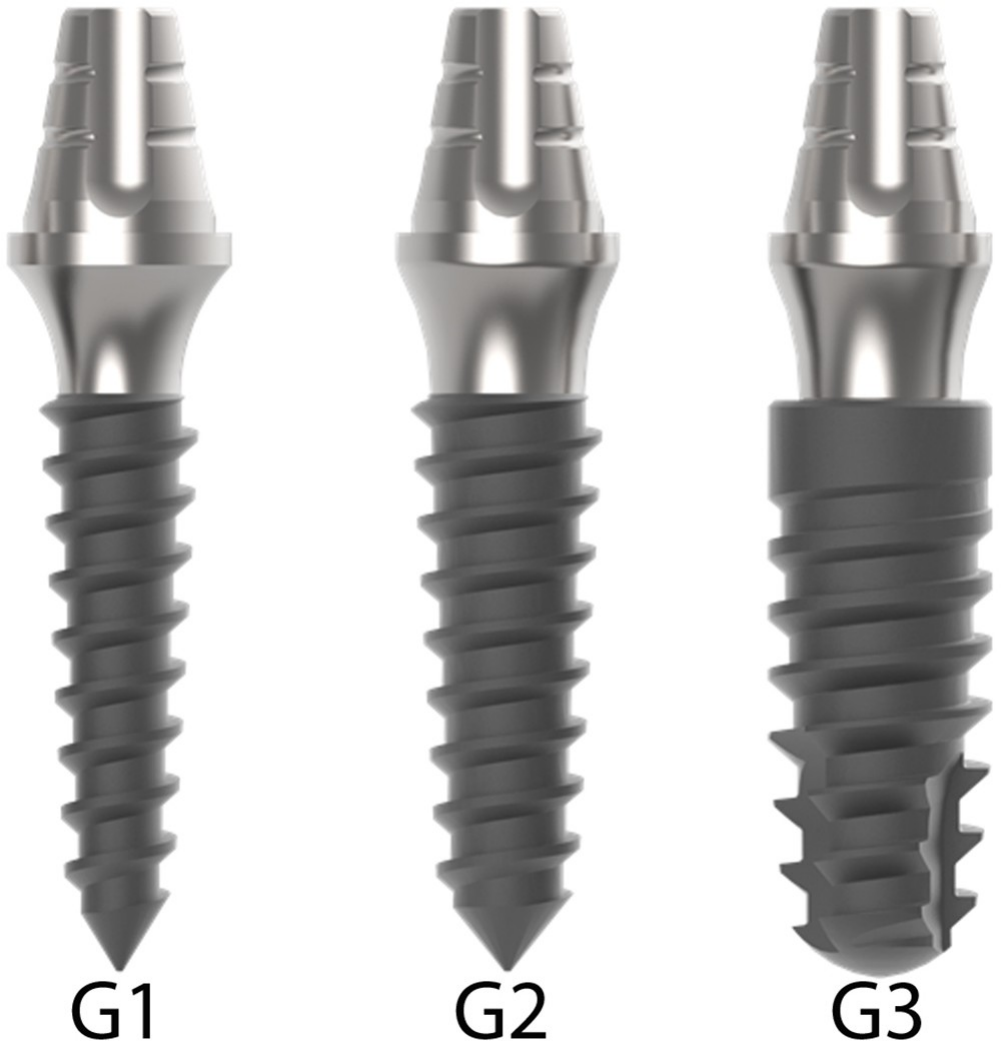
Representative image of the implants tested in each group. The groups G1 and G2 are one-piece implants and the group G3 is two-pieces implant.

In the present study, two diameters of one-piece implants and a narrow-diameter two-piece implant were used, forming three groups: Group G1, where one-piece implants with an extra-narrow diameter of 2.5 mm were used; Group G2, where one-piece implants with a narrow diameter of 3.0 mm were used; Group G3, where two-piece implants with a narrow diameter of 3.5 mm with a Morse taper connection were used. The smaller two-piece diameter implant available in this implant system was used to serve as a comparison parameter. All implants used were 9.0 mm in length from the implantable portion and 6 mm in length by 3.5 mm in diameter from the portion corresponding to the abutment. In addition, all abutments had a 2.5 mm transmucosal portion. All implants and abutments used are manufactured by the company Implacil De Bortoli (São Paulo, Brazil). Fig 1 shows the sets (implant and abutment) tested in each group.

The digital models were made using the program Rhinoceros 5.4.1 for Windows (Robert McNeel and Associates, Seattle, USA). In this stage, we digitally designed the macrogeometry of the bone base, prosthetic crown and cement. The geometries of the implants, abutments and copings were provided by the manufacturer (Implacil De Bortoli, São Paulo, Brazil). In the second stage, processing was performed using the software Ansys Workbench 19.0 (Ansys Inc., Canonsburg, PA, EUA). Finally, an analysis of the qualitative and quantitative results was carried out through the evaluation of numerical and graphical information regarding the stress values of each component of the three-dimensional model.

**Fig 2:**
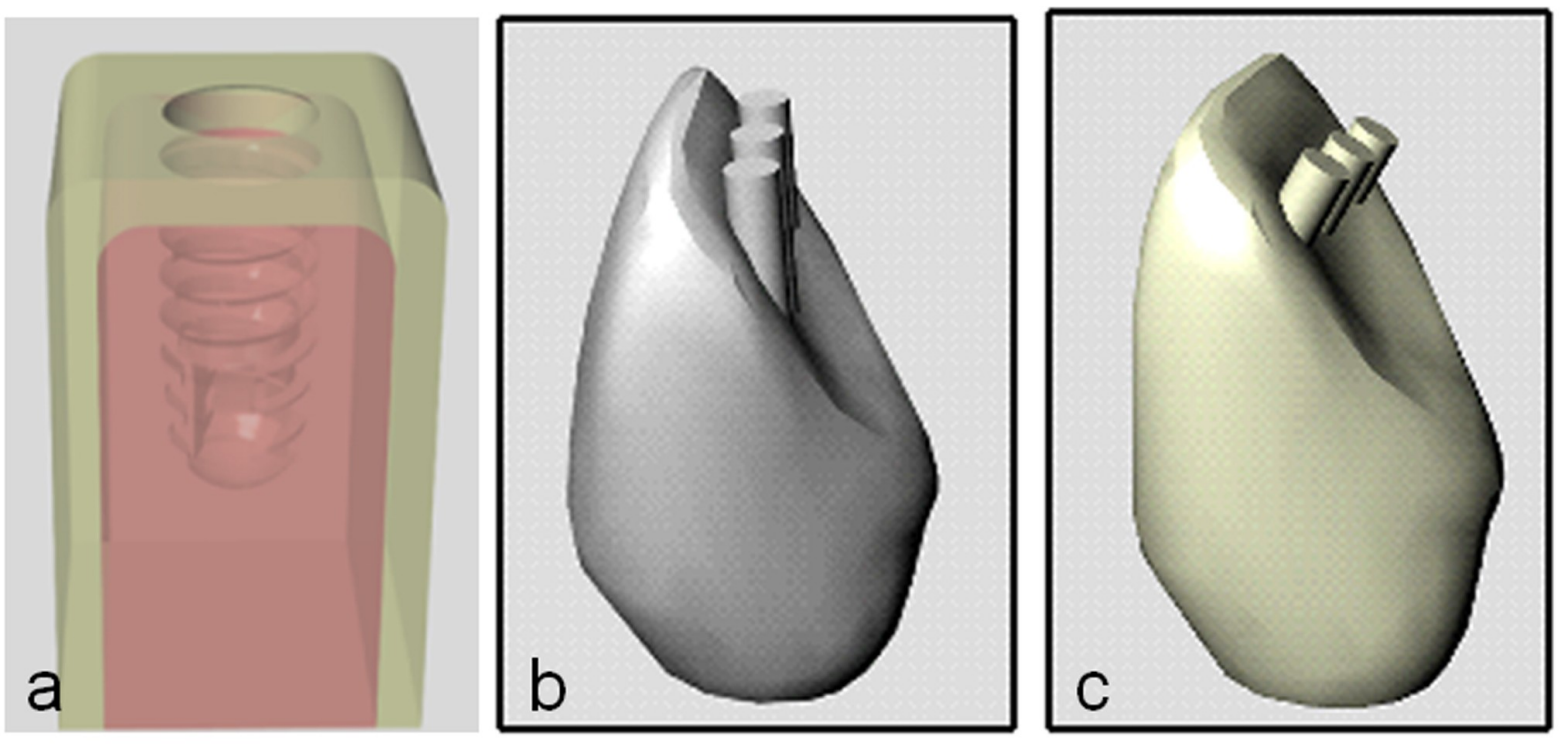
Image of the structures created to test the samples of each group. (a) bone block, (b) crown for axial load application and (c) crown for angled load application in 30°.

The design of alveolar bone base was performed with the cortical bone portion at 1.0 mm of thickness [11]. The designs of the prosthetic crowns were modeled in the program Rhinoceros 5.4.1 for Windows (Robert McNeel and Associates, Seattle, USA) and standardized as unitary geometric structures of feldspathic porcelain crowns corresponding to an upper lateral incisor. On top of the crowns, cylindrical structures were inserted in a direction parallel to the implant (axial load) and, in another implant, with a 30° inclination in relation to the long axis of the implant sets (angled load). The cylindrical structures were inserted in the crowns to assure the application of load in the same position for all implant models. As the teeth are organic geometric structures, in order to have uniformity and correct angulation control, we use this methodology for exact standardization of the axial and oblique load in all proposed models. Fig 2 show the alveolar bone and both crown designs.

The assembly of the clinical situation models (bone base, implant, abutment and crown) was also performed using the Rhinoceros program 5.4.1 for Windows (Robert McNeel and Associates, Seattle, USA) and analyzed in the Ansys Workbench 19.0 software (Ansys Inc., Canonsburg, PA, EUA). For each material of the modeled structures (crown, metallic infrastructure, cement, abutment, implant, cortical bone and cancellous bone), values corresponding to the mechanical properties of each element were inserted individually (modulus of elasticity and Poisson’s ratio), as specified in Table 1. All materials were considered to be isotropic, homogeneous and linearly elastic. For the G3 group (two-piece implant), a non-linear analysis was carried out, assuming the frictional nature of the Morse connection between the sets (abutment-implant-fixation screw).

**Table 1:** Mechanical properties of the materials used in the present study.

Materials	Young´s modulus (GPa)	Poisson ratio	References
Feldspathic porcelain	65	0.33	Álvarez et al. [12]
Metallic infrastructure	218	0.25	Toniollo et al. [13]
Cement	7	0.30	Álvarez et al. [12]
Titanium abutment	107.2	0.35	Álvarez et al. [12]
Titanium implant	110	0.25	Geng et al. [14]
Cancellous bone	1.37	0.30	Geng et al. [14]
Cortical bone	15	0.33	Álvarez et al. [12]

**Fig 3:**
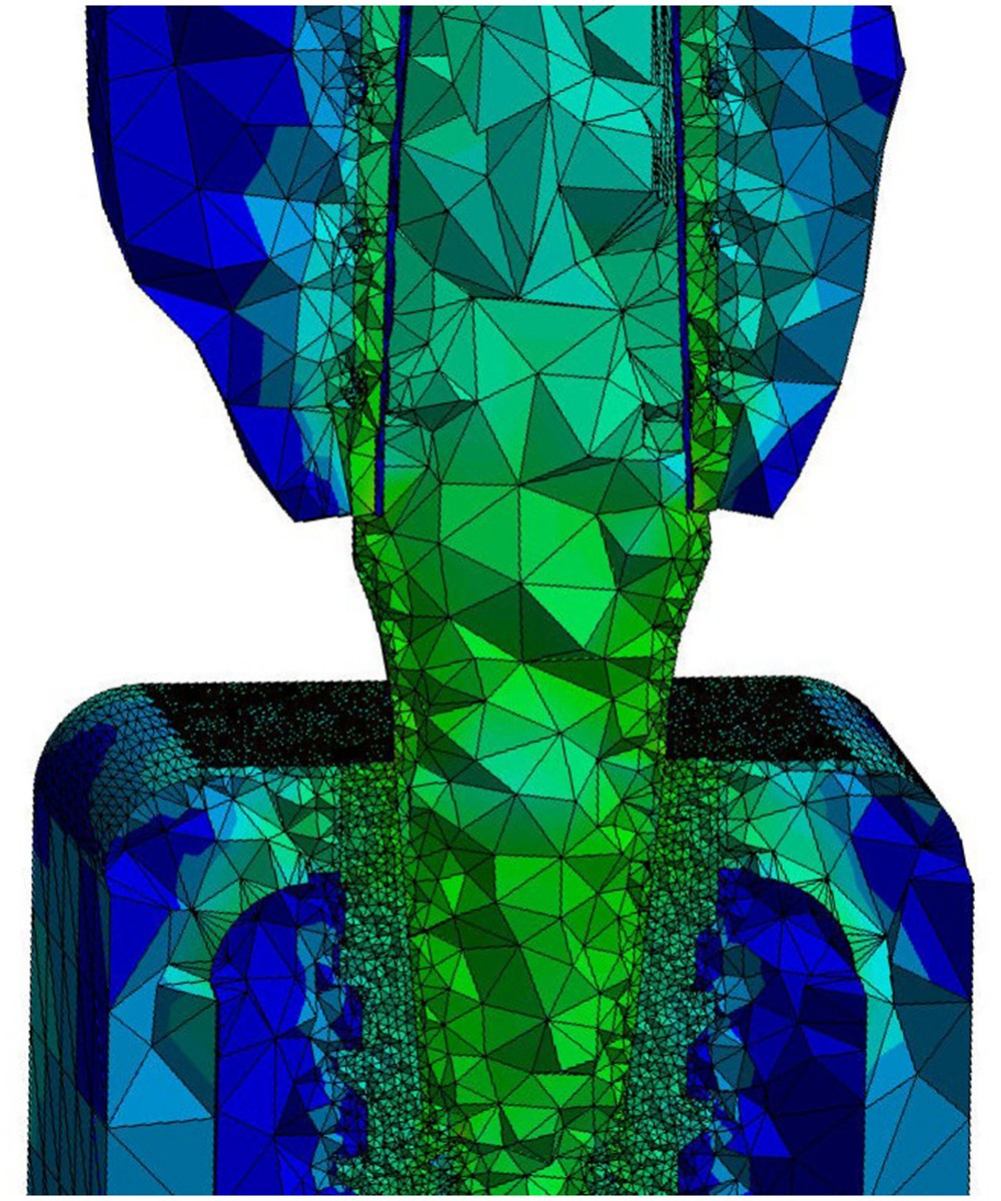
Mesh image generated for the structures to be studied, showing that the regions corresponding to the areas of more interest for the present study, the mesh refinement was carried out.

A mesh refinement was carried out for the regions corresponding to the areas of more interest for the present study (Fig 3). The total number of elements in the models analyzed ranged from 432.020 to 887.245, and the total number of nodes ranged from 753.005 to 1398.103.

In relation to the simulation limits, the contacts between the fundamental structures of the geometric model were considered to be bonded contact between the crown and metallic infrastructure, between metallic infrastructure and cement, between cement and abutment, between cancellous bone and cortical bone and between the implant and bone. A structure with a minimum thickness of up to 0.1 mm of resin cement was built. Between the internal surface of the implant and the external surface of the abutment, a friction type contact was adopted, using a friction coefficient of 0.8 [14]. The bone block was considered fixed in its location in the mesial and distal areas of the block. In all created situations, a force of 150 N was applied to the cylindrical surfaces drawn on the crowns [15,16].

All simulations in this study were analyzed quantitatively and qualitatively. The results were individualized according to the main structures of interest in the study: implant, abutment and peri-implant bone (cortical bone and cancellous bone). The prosthetic crown, metallic infrastructure and cement were modeled and inserted in the simulations just to simulate a clinical situation; therefore, the quantitative and qualitative results of these structures were not analyzed.

All implant models and peri-implant bone (cortical and cancellous bone) were analyzed using the von Mises criterion. The Von Mises criterion is one of the most used criteria in dentistry to assess bone performance, as it has the advantage of always providing a positive result, facilitating comparative analyzes. However, as the Von Mises criterion is most appropriate to ductile structures [17–19], to circumvent this limitation, we associate the Mohr-Coulomb method to analyze the bone structure [20,21]. Then, the peri-implant bone was also subjected to the Mohr–Coulomb method, which allows the differentiation of the impact of tension and compression stresses to occur in a different way. In evaluating the flow limits of each structure, the values considered were as follows: for the titanium implant, the value of 1130 MPa corresponds to 100% [22] and, for bone tissue, 114 Mpa corresponds to 100% in situations of axial load application, and 50 Mpa corresponds to 100% in the application of angled loads [23].

## Results

### Axial load results

**Fig 4:**
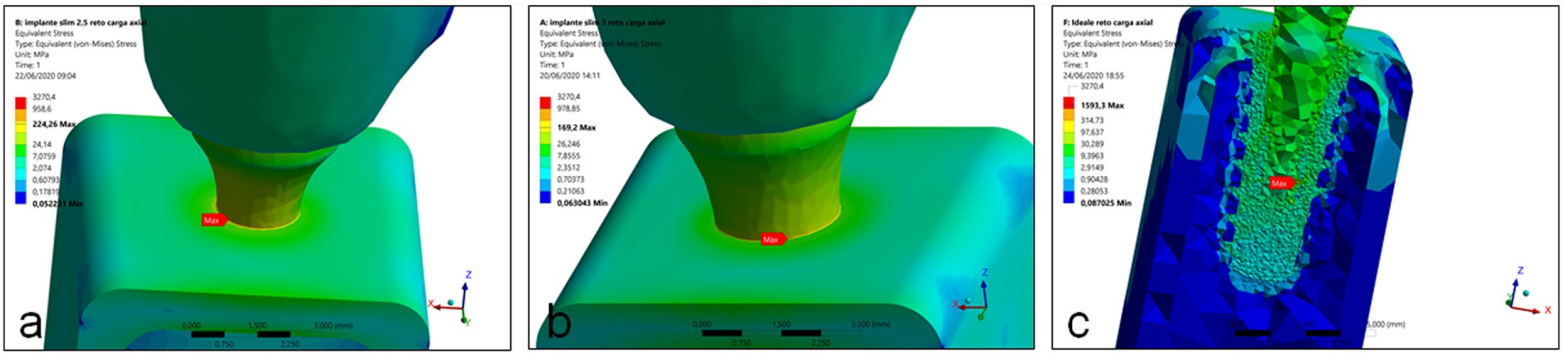
Images showing the position of the maximum stress of implant structure in each group during the axial load application. (a) group G1, (b) group G2 and (c) group G3.

In the G1 and G2 groups, during the application of an axial load, the maximum von Mises stress values to the implant structure was observed in the cervical portion, corresponding to the cervical cortical bone level around the implant. Whereas in the group G3, the maximum von Mises stress values was observed inside of the implant cone, corresponding to the abutment position, with low incidence in bone tissue. Fig 4 show the position of the maximum stress in each group. The highest value of maximum stress was observed in the implant of the group G3 (1593.3 MPa), followed by the group G1 (224.26 MPa) and the group G2 (169.2 MPa). In the evaluation of the maximum stress value for the perimplant bone, the 3 groups presented low values in the cancellous bone (3 to 12 MPa). However, in the cortical bone it was where the measured stress values were highest, and in the group G1 it was the highest of all, which was 22.35% higher than that of group G2 and 321.23% than the group G3. Table 2 shows the minimum and maximum von Mises stress values found for the axial load for each group.

**Table 2:** Minimum and maximum von Mises principal stress of implants with different structures with axial load direction (values in MPa).

Groups	Implants		Cortical bone		Cancellous bone	
	σmin	τmax	σmin	τmax	σmin	τmax
**G1**	0.052	224.26	0.052	104.55	0.075	12.051
**G2**	0.063	169.2	0.063	85.45	0.103	9.152
**G3**	0.087	1593.3	0.112	24.82	0.304	3.286

σmin = minimum principal stress; τmax = maximum shear stress.

**Fig 5:**
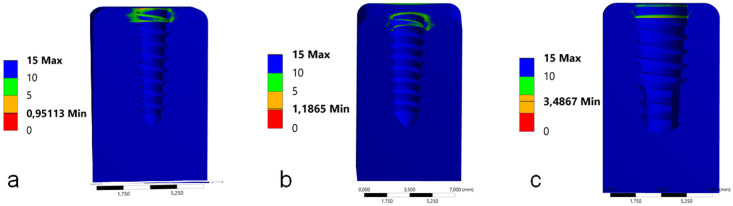
Images with the values of the Mohr-Coulomb analysis of peri-implant bone for each group during the axial load application. (a) group G1, (b) group G2 and (c) group G3.

In the Mohr-Coulomb analysis, the lower the values obtained, the more harmful the peri-implant bone. Then, in this analysis the group G1 showed the less values, followed by the group G2 and G3, as presented in the Fig 5.

### Angled 30° load results

**Fig 6:**
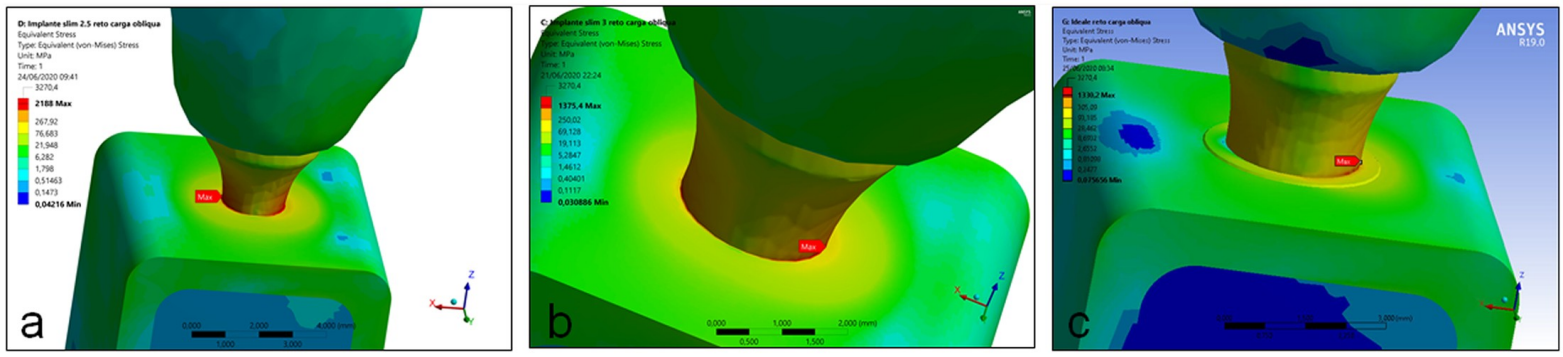
Images showing the position of the maximum stress of implant structure in each group during the angled load application. (a) group G1, (b) group G2 and (c) group G3.

In the application of an angled load, the maximum von Mises stress values to the implant structure in all groups was observed in the cervical portion. However, in the groups G1 and G3 the maximum stress was observed directly in the interface (bone-to-implant) of the cervical portion, corresponding to the cervical cortical bone level around the implant. Whereas in the group G3, the maximum von Mises stress values was observed in the cervical portion of the abutment in contact with the implant platform, with low incidence in cortical bone. Fig 6 show the position of the maximum stress in each group. The maximum stress value presented in the group G1 was 59% highest than the group G2 and 64.5% highest than the group G3. In the evaluation of the maximum stress value for the perimplant bone, the 3 groups presented low values in the cancellous bone between 12 to 24 MPa (G1 > G2 > G3). In the cortical bone were found highest stress values between the groups, with the groups G1 and G2 showing a similar value (# 3.5%) and a highest difference for the group G3 (391.8%). Table 3 shows the minimum and maximum von Mises stress values found for the angled load for each group.

**Table 3:** Maximum von Mises stresses of implants with different structures with angled load direction (values in MPa).

Groups	Implants		Cortical bone		Cancellous bone	
	σmin	τmax	σmin	τmax	σmin	τmax
**G1**	0.042	2188	0.042	607.9	0.042	24.077
**G2**	1.901	1375.4	0.031	587.47	0.030	22.176
**G3**	0.076	1330.2	0.076	123.6	0.075	12.581

σmin = minimum principal stress; τmax = maximum shear stress.

**Fig 7:**
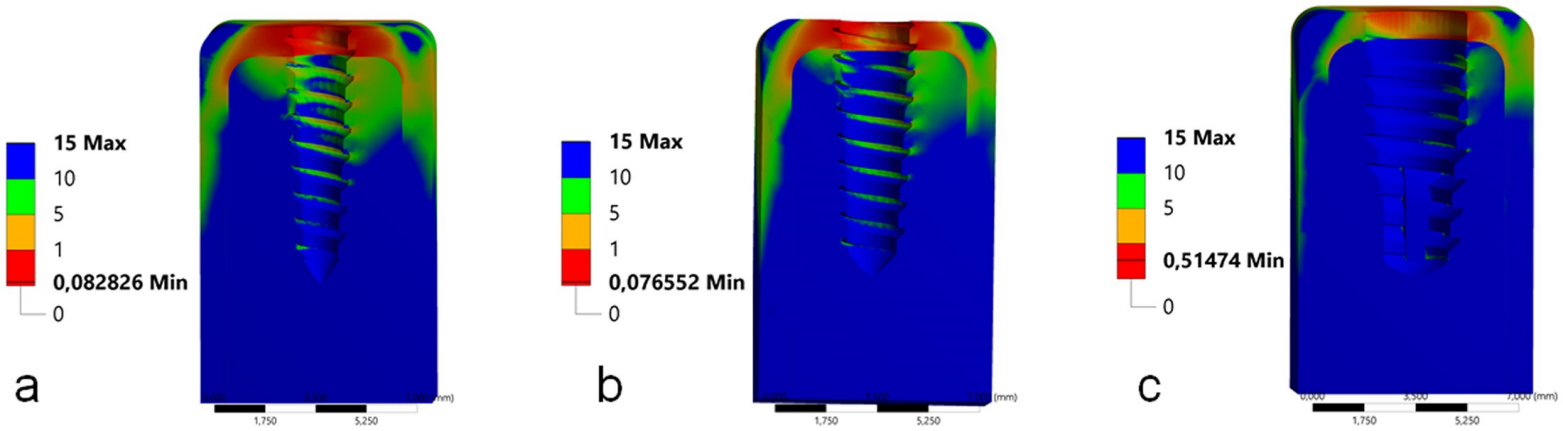
Images with the values of the Mohr-Coulomb analysis of peri-implant bone for each group during the angled load application. (a) group G1, (b) group G2 and (c) group G3.

In the Mohr-Coulomb analysis, the group G2 showed the less values, followed by the group G1 and G3, as presented in the Fig 7.

## Discussion

Our in vitro study aimed to analyze the stress distribution in three models of in one- and two-piece dental implants with reduced diameters by applying a 150 N load in the axial and angled directions. In the application of the axial load, all tested models presented stress values below the yield limits of the analyzed structures. However, when angled loads were applied, the behavior was different between the groups, with values above the flow limit in the implant structure and in the supporting bone tissue. As presented and considered in other studies [24,25], in our study, the materials were modeled with linear, elastic, isotropic and homogeneous properties. The finite element analysis methodology used is often applied to the type of evaluation reported in the present study, as it presents a high level of reliability that potentially reaches 95% [26,27]. Through this methodology, it was possible to carry out a quantitative analysis and a qualitative analysis of the simulated loads, which gives us an idea of the way in which the applied loads are dissipated and the possible consequences in all evaluated structures. In addition, an analysis was performed for peri-implant bone using the Mohr–Coulomb criterion, which is more suitable for bone tissue, as it takes into account the criteria of traction and compression of a material [21].

Regardless of the methodology and implants used, the results of our study corroborate the results reported by other studies [28–31], which demonstrated that the area of the cortical bone crest is always more exposed to harmful forces, especially when non-axial loads are applied. The concern with the dissipation of loads in the region of the marginal ridge is a constant in several studies found in the literature since stress concentrations above the flow limit can cause microfractures in the cortical bone area and induce bone resorption. In addition, our results corroborate other studies [5,21,27,28,32,33] that demonstrated that the regions with the highest concentration are the neck area and the region of the first threads, and therefore, these would be better studied in one-piece implants, especially in those with an extra-narrow diameter.

Other authors [21,28,34–36] have evaluated the behavior of implants with different connections and observed that they presented a superior mechanical behavior when compared with hexagonal connections (internal and external), demonstrating a lower tension under oblique forces and the superior dissipation capacity in implants with Morse taper connections, even under angled loads. For this reason, a two-piece implant and reduced platform with a Morse taper connection were selected for our comparison. However, authors who compared one- and two-piece (Morse taper connection) implants of the same diameter reported divergent results [5,37]. Wu and collaborators [5] showed favorable results for two-piece implants, while Lopes and collaborators [37] demonstrated better results for one-piece implants. Our results show that Morse taper implants (two-pieces) produce better results in relation to the dissipation of forces applied in both directions (axial and angled), mainly in the evaluation of the peri-implant region. The conical shape of the Morse taper connection causes most of the forces received to be dissipated into the implant.

Unsurprisingly, in studying different implant diameters, several authors [38–41] found better performances with the use of larger-diameter implants. The results presented in our study corroborate those obtained by other authors [4,42,43], who also found a good mechanical behavior of single-body implants, regardless of the material from which they were manufactured, concluding that small-diameter implants can be used in implant-supported rehabilitation with success and predictability rates similar to implants with a conventional diameter.

When comparing the behavior of small-diameter single-body implants with two-piece implants of different diameters, our results reinforce the findings of other authors [3,44], who stated that despite the better behavior of regular-diameter implants, small-diameter implants are a viable and predictable option for implant-supported rehabilitation, provided that careful planning is carried out. Still, other authors [45–47] verified a decrease in the mechanical resistance of extra-reduced-diameter implants when compared with regular-diameter implants, and for this reason, their clinical indication would be restricted to areas with a low incidence of masticatory loads.

Although the maximum values of Von Mises stresses exceeded the deformation limit of the extra-narrow implant when subjected to an angled load, in the other groups tested, only a few points on the implant body or abutment body (two-piece implants) exceeded flow values, corroborating the study of Bordin and collaborators [1] who, through finite element analysis, demonstrated the good behavior of small-diameter implants. The results of the present study are reinforced by the findings of Cinel and collaborators [33], who demonstrated the efficiency of small-diameter single-body implants. However, Cinel and collaborators [33] pointed out a greater tension in some areas and possible failure due to fatigue, which diverges from the present study in this aspect because, according to the results obtained in this work, these concentration peaks found in cortical areas and in the first turns are located in well-defined areas, so they are not considered points of possible fatigue failures.

Due to the large number of variables existing in each patient and among patients, it becomes impossible to accurately reproduce clinical behavior in vitro. Then, some limitations of the present study can be related: this mode of analysis by finite elements does not allow to consider the influence of the forces applied by the tongue and other structures present in the oral cavity, as well as, the influence of the different load conditions present during the masticatory cycle, that is, the applied loads were in a single predetermined direction; the jawbone was considered to be isotropic and homogenous, and the interface between cortical/cancellous bone and between the implant and cortical/cancellous bone has been assumed completely bonded, although this is not the case in clinical conditions; the occlusal forces have been applied in a predeterminate position of the crown, while, in real clinical conditions these are applied in different positions.

One-piece narrow-diameter implants demonstrate a promising alternative to avoid previous procedures (grafts and/or orthodontic movement) brings an immediate benefit to the solution of the case. However, clinicians should consider other clinical parameters, such as clinical occlusion, parafunctional habits, the position of the site and, mainly, the direction of the loads that will fall on the crown. Thus, implants with extra-small diameter can and must be protected from angled loads. This is possible, as they are supposed to be positioned between healthy teeth. Furthermore, an in vitro study to support the computational models is subsequently employed to verify the in silico results (FEA analysis) are meant to show how materials behave and not be used for quantitative experimental data.

## Conclusions

Within the limitations of the present study, we conclude that the stress distribution on axial and angled 30° loads on the peri-implant bone tissue presented a better result in the two-pieces narrow implant, in comparison with the one-piece narrow implants. Moreover, the maximum stress values obtained for the implant structure in angled load was well above the limit of resistance in the one-piece extra-narrow implants.
